# Predictors for depressive symptoms by four types of disability

**DOI:** 10.1038/s41598-021-98765-4

**Published:** 2021-09-29

**Authors:** Sun Wook Jung, Jin-Ha Yoon, Wanhyung Lee

**Affiliations:** 1 Samsung Medical Center, Seoul, Republic of Korea; 2 Department of Preventive Medicine, Yonsei University College of Medicine, Seoul, Republic of Korea; 3 The Institute for Occupational Health, Yonsei University College of Medicine, Seoul, Republic of Korea; 4 Department of Occupational and Environmental Medicine, Gil Medical Center, Gachon University College of Medicine, 21, Namdong-daero 774, Incheon, 21565 Republic of Korea

**Keywords:** Risk factors, Diseases, Psychiatric disorders

## Abstract

This study aimed to examine the longitudinal relationship between disability and depressive symptoms, by comparing four types of disability in community-dwelling individuals with disabilities in South Korea. A total of 3347 South Koreans with disabilities from the second wave of the Panel Survey of Employment for the Disabled was utilized. Depressive symptomatology was assessed by whether the participant had experienced depressive symptoms for more than two weeks during the past year. A multivariate logistic regression model was used to calculate the odds ratio (OR) for depressive symptoms, and a Cox proportional hazards model to calculate the hazard ratio (HR) for two-year survival analysis. Persons who acquired mental disability from accident or industrial disaster and persons with congenital physical-internal disability were at higher risk for depressive symptoms. Maintaining employment was found to be an effective way to decrease the risk of depressive symptoms in persons with physical-external disability, sensory/speech disability, or mental disability. In contrast, in physical-internal disability, retaining normal ability to work seemed to be the key to reduce the risk of depressive symptoms. Predictors of depressive symptoms were found to differ depending on the type of disability. Such differences should be reflected in clinical and policy-level interventions to address the specific psychiatric needs of persons with different disabilities.

## Introduction

According to the World Health Organization (WHO), disability refers to difficulties in human functioning—including body function, executing activities, or involvement in any area of life^1^. Disability is a core public health problem not only because it affects a large number of population worldwide, but also because most people will experience disability at some point in life^1^. More than one billion, or 15% of the world’s population, are living with disabilities^2^, and the global trends in aging populations with higher risk of disability are likely to further increase in prevalence^3^.

Depression is one of the most common secondary conditions associated with disability. As constructed through dynamic interactions between health conditions and contextual (i.e., environmental and personal) factors, disability profoundly impacts an individual’s mental wellbeing^4^. Abundant research has demonstrated that people with disabilities are at higher risk of depressive symptoms than the general population in both men and women of all ages, as a consequence of biological mechanisms, as well as social factors such as loss of independence in daily living and restricted social participation^5–8^. For instance, two longitudinal studies respectively indicated that blindness and severe hearing impairment elevated the risk of depression in these individuals^9,10^. Another review study found that the risk of depression was higher in populations with spinal cord injuries, multiple sclerosis, and intellectual disabilities^7^.

Despite the wealth of literature on the association between disability and depression, the cross-sectional nature of most studies has prevented any inference of the direction of the relationship between factors associated with disability and depression. Recent research suggests that impact of disability on depressive symptoms with longitudinal study^11^. Few have compared all types of disabilities to reveal different key factors contributing to the onset or progression of depression. Previous studies on disability and depression have mostly focused on one or several specific types of disabilities, failing to ensure to an extent which the results can be generalized and applied to other types of disabilities. However, disability is a heterogeneous term, which encompasses the visible parts of body to the invisible internal organs or mental functions; and from congenital conditions to acquired injuries or diseases^1^. Moreover, each type of disability has different dynamics in the interaction between health conditions and contextual factors such as personal factors (e.g., gender, age, socioeconomic status) and environmental factors (e.g., independence or ability to self-care, employment status, social isolation, or discrimination)^12^.

In order to examine the causal mechanisms of disability leading to depression, it is important to investigate the diversity and heterogeneity of disability, as well as the common features shared by various types of disability through a longitudinal analysis. To the best of our knowledge, this is the first epidemiological study to compare all types of disability in terms of the causal relationship between disability and depressive symptoms. Each type of disability accompanies different kinds of difficulties or limitations in human functions. For instance, impairments in the external part of the body can lead to appearance concerns, while sensory impairments can lead to restrictions in communication or leisure activities such as the enjoyment of art. In addition, the same type of disability may result in different disability experiences depending on the cause of disability, environmental factors, or personal factors. This is where a study that reveals the shared and unshared features of various disability categories can add value.

This study aims to fill the knowledge gaps by examining the causal relationship between disability and depressive symptoms, through the stratification by four disability types using a two-year follow-up study of community-dwelling individuals with disabilities in South Korea. In terms of contextual factors, this study focused more on environmental factors than personal factors since these are improvable by clinical or policy-level interventions. We expect that the findings of this study may strengthen the theoretical foundation for mental health interventions for persons with disabilities.

## Results

Table 1 summarizes the general characteristics of study participants stratified by depressive symptomatology. At baseline, the prevalence of depressive symptoms was 17.7% (n = 727) for the total number of participants in the first year. Among the four disability categories, physical-external disability was 56.4% (n = 2139), sensory/ speech was 22.1% (n = 909), physical-internal was 8.0% (n = 328), and mental was 13.5% (n = 553) of the total at baseline. The prevalence of depressive symptoms was higher in the physical-internal disability and mental disability categories (23.8% and 23.2%, respectively) than the physical-external disability and sensory/speech disability categories (16.6% and 15.0%, respectively). During the two-year follow-up, the prevalence of depressive symptoms was 16.2% (n = 449) for the cohort that excluded participants with depressive symptoms at baseline. The prevalence of depressive symptoms was the highest in those with physical-internal disability (25.0%, n = 52), and the lowest in those with physical-external disability (14.7%, n = 238). At baseline, those who were women, of older age, living without a partner, earning a lower household income, of a smaller household size, living in the metropolitan area, unemployed, unable to work, of low social activity participation, in need of assistance in daily living, discriminated against, of low friendship satisfaction, of low leisure satisfaction, diagnosed with chronic conditions, and current smokers were found to have a higher prevalence of depressive symptoms. This was mostly consistent in the two-year follow-up of participants without depressive symptoms, except that there was no evidence of association between depressive symptoms and age (p = 0.17), area of residence (p = 0.49), and smoking status (p = 0.1) at the two-year follow-up.

**Table 1:** General characteristics and the prevalence of depressive symptoms of the participants at baseline and the two-year follow up of the participants without depressive symptom.

Variable	Total participants (1st year)		
	Symptom ( +)	No symptom	Total
**Type of disability**			(*P*-value) < 0.001*
Physical-external	385 (16.6)	1934 (83.4)	2319 (100.0)
Sensory/speech	136 (15.0)	773 (85.0)	909 (100.0)
Physical-internal	78 (23.8)	250 (76.2)	328 (100.0)
Mental	128 (23.2)	425 (76.9)	553 (100.0)
**Cause of disability**			< 0.001*
Congenital/intranatal cause	75 (12.5)	525 (87.5)	600 (100.0)
Accident/industrial disaster	237 (15.8)	1265 (84.2)	1502 (100.0)
Disease	278 (20.0)	1115 (80.0)	1393 (100.0)
Unknown cause	137 (22.3)	477 (77.7)	614 (100.0)
**Gender**			0.02*
Male	448 (16.7)	2242 (83.4)	2690 (100.0)
Female	279 (19.7)	1140 (80.3)	1419 (100.0)
**Age**			< 0.001*
< 35	168 (14.2)	1018 (85.8)	1186 (100.0)
35–44	173 (15.2)	964 (84.8)	1137 (100.0)
45–54	213 (21.0)	800 (79.0)	1013 (100.0)
> 55	173 (22.4)	600 (77.6)	773 (100.0)
**Marital status**			< 0.001*
Married/ cohabiting	220 (12.0)	1614 (88.0)	1834 (100.0)
Single/divorced/widowed	507 (22.3)	1768 (77.7)	2275 (100.0)
**Household income**			< 0.001*
< 10	337 (27.3)	899 (72.7)	1236 (100.0)
10–25	197 (17.9)	904 (82.1)	1101 (100.0)
25–40	101 (12.6)	703 (87.4)	804 (100.0)
> 40	92 ( 9.5)	876 (90.5)	968 (100.0)
**Household size**			< 0.001*
1	218 (27.1)	586 (72.9)	804 (100.0)
2	207 (20.9)	782 (79.1)	989 (100.0)
3	142 (14.4)	847 (85.6)	989 (100.0)
4 or more	160 (12.1)	1167 (87.9)	1327 (100.0)
**Area of residence**			0.024*
Non-metropolitan	207 (15.7)	1111 (84.3)	1318 (100.0)
Metropolitan	520 (18.6)	2271 (81.4)	2791 (100.0)
**Employment status**			< 0.001*
Employed	211 (10.4)	1816 (89.6)	2027 (100.0)
Unemployed	516 (24.8)	1566 (75.2)	2082 (100.0)
**Occupational ability**			< 0.001*
Normal	229 (10.7)	1920 (89.3)	2149 (100.0)
Lower but able to work	199 (19.7)	810 (80.3)	1009 (100.0)
Unable to work	299 (31.4)	652 (68.6)	951 (100.0)
**Social activity participation**			< 0.001*
High	188 ( 9.6)	1768 (90.4)	1956 (100.0)
Low	539 (25.0)	1614 (75.0)	2153 (100.0)
**Assistance in daily living**			< 0.001*
Not/ rarely needed	367 (13.6)	2334 (86.4)	2701 (100.0)
Needed	360 (25.6)	1048 (74.4)	1408 (100.0)
**Discrimination due to disability**			< 0.001*
Rare	433 (14.1)	2640 (85.9)	3073 (100.0)
Common	294 (28.4)	742 (71.6)	1036 (100.0)
**Friendship satisfaction**			< 0.001*
Satisfied	254 (10.1)	2260 (89.9)	2514 (100.0)
Not satisfied	473 (29.7)	1122 (70.3)	1595 (100.0)
**Leisure satisfaction**			< 0.001*
Satisfied	125 ( 8.8)	1302 (91.2)	1427 (100.0)
Not satisfied	602 (22.5)	2080 (77.6)	2682 (100.0)
**Chronic conditions**			< 0.001*
No	443 (14.5)	2615 (85.5)	3058 (100.0)
Yes	284 (27.0)	767 (73.0)	1051 (100.0)
**Smoking**			0.003*
Non-smoker	537 (16.7)	2671 (83.3)	3208 (100.0)
Current smoker	190 (21.1)	711 (78.9)	901 (100.0)

Table 2 displays the results from the cross-sectional analysis of the total participants and the two-year follow-up analysis of the participants without depressive symptoms at baseline to assess the link between disability and depressive symptoms. At baseline, the crude OR for depressive symptoms was 1.57 in the physical-internal disability (95% CI 1.18–2.06) and 1.51 in mental disability (95% CI 1.20–1.89) categories compared to physical-external disability, although after adjustment there was no evidence of association between depressive symptoms and both physical-internal disability (95% CI 0.90–1.71) and mental disability (95% CI 0.60–1.06). In the two-year follow-up, the crude HR for depressive symptoms was 1.78 (1.32–2.41) for those with mental disability, whereas the HR became 0.69 (95% CI 0.50–0.95) after adjustment. Disability by unknown cause and disability by disease showed higher ORs (1.75, 95% CI 1.33–2.31; 2.01, 95% CI 1.48–2.74) than disability by congenital/intranatal cause, and the associations were not attenuated after adjusting for other covariates. The OR for depressive symptoms was 1.34 in those who were unemployed (95% CI 1.05–1.71) compared to those who were employed; 1.65 for those with low social activity participation (95% CI 1.35–2.03) compared to those with high social participation; 1.34 in those in need of assistance in daily living (95% CI 1.10–1.64) compared to those who were independent; 1.67 in those who were discriminated against (95% CI 1.37–2.03) compared to those who were rarely discriminated against; 1.89 in those who were not satisfied with their friendships (95% CI 1.55–2.30) compared to those who satisfied; and 1.72 in those who were notsatisfied with leisure (95% CI 1.37–2.16) compared to those satisfied. In the two-year follow-up, the HR was 1.99 (95% CI 1.54–2.57) for those who were unemployed, 1.38 (1.12–1.71) in those with low social participation, 1.69 (95% CI 1.36–2.09) in those discriminated against, and 1.35 (95% CI 1.08–1.69) in those who were satisfied with leisure. Additionally, there was no evidence that occupational ability (95% CI 0.84–1.41; 0.80–1.45), assistance in daily living (95% CI 0.83–1.28), or friendship satisfaction (95% CI 0.97–1.48) may affect depressive symptoms.

**Table 2:** A cross-sectional (at baseline of total participants) and a longitudinal (two-year follow up of participants without depressive symptom) analysis on the crude and adjusted hazard ratios (95% CI) for depressive symptoms.

	Cross-sectional analysis of the total subjects (1st year)		Two year follow-up of the subjects with no depressive symptom	
	Crude	Adjusted^†^	Crude	Adjusted^†^
**Type of disability**				
Physical-external	1.00	1.00	1.00	1.00
Sensory/speech	0.88 (0.71–1.09)	0.83 (0.67–1.08)	1.04 (0.82–1.31)	0.98 (0.77–1.25)
Physical-internal	1.57 (1.18–2.06)*	1.24 (0.90–1.71)	1.78 (1.32–2.41)*	1.39 (1.01–1.92)*
Mental	1.51 (1.20–1.89)*	0.80 (0.60–1.06)	1.37 (1.04–1.82)*	0.69 (0.50–0.95)*
**Cause of disability**				
Congenital/intranatal cause	1.00	1.00	1.00	1.00
Accident/industrial disaster	1.31 (1.00–1.74)	1.59 (1.16–2.20)*	0.75 (0.56–1.00)*	1.01 (0.74–1.39)
Disease	1.75 (1.33–2.31)*	1.68 (1.24–2.30)*	1.09 (0.82–1.43)	1.15 (0.85–1.54)
Unknown cause	2.01 (1.48–2.74 )*	1.80 (1.30–2.52)*	1.19 (0.86–1.65)	1.16 (0.83–1.62)
**Employment status**				
Employed	1.00	1.00	1.00	1.00
Unemployed	2.84 (2.39–3.38)*	1.34 (1.05–1.71)*	2.93 ( 2.39–3.58)*	1.99 (1.54–2.57)*
**Occupational ability**				
Normal	1.00	1.00	1.00	1.00
Lower but able to work	2.06 (1.67–2.53)*	1.03 (0.80–1.32)	1.87 (1.49–2.35)*	1.09 (0.84–1.41)
Unable to work	3.84 (3.17–4.67)*	1.19 (0.89–1.58)	2.71 (2.17–3.37)*	1.08 (0.80–1.45)
**Social activity participation**				
High	1.00	1.00	1.00	1.00
Low	3.14 (2.63–3.76)*	1.65 (1.35–2.03)*	2.07 (1.71–2.51)*	1.38 (1.12–1.71)*
**Assistance in daily living**				
Not/ rarely needed	1.00	1.00	1.00	1.00
Needed	2.18 (1.86–2.57)*	1.34 (1.10–1.64)*	1.66 (1.38–2.01)*	1.03 (0.83–1.28)
**Discrimination due to disability**				
Rare	1.00	1.00	1.00	1.00
Common	2.42 (2.04–2.86)*	1.67 (1.37–2.03)*	2.10 (1.73–2.55)*	1.69 (1.36–2.09)*
**Friendship satisfaction**				
Satisfied	1.00	1.00	1.00	1.00
Not satisfied	3.75 (3.17–4.44)*	1.89 (1.55–2.30)*	2.00 (1.66–2.40)*	1.20 (0.97–1.48)
**Leisure satisfaction**				
Satisfied	1.00	1.00	1.00	1.00
Not satisfied	3.01 (2.46–3.71)*	1.72 (1.37–2.16)*	1.75 (1.42–2.16)*	1.35 (1.08–1.69)*

^†^Adjusted for type of disability, cause of disability, gender, age, marital status, household income, household size, area of residence, employment status, occupational ability, social activity participation, assistance in daily living, discrimination due to disability, friendship satisfaction, leisure satisfaction, chronic conditions, and smoking. During each stratification analysis, factors that were stratified for were not included in the adjustment.

Table 3 shows the results from the two-year prospective analysis of crude and adjusted HRs stratified by four types of disability. For physical-external disability, cause of disability, employment status, occupational ability, social activity participation, assistance in daily living, discrimination, friendship satisfaction, and leisure satisfaction had longitudinal associations with depressive symptoms in the crude model. The crude HRs were 0.47 for disability by accident/industrial disaster (0.32–0.67) and 0.68 in disability by unknown cause (0.46–0.99), although such longitudinal association no longer existed after adjustment. However, unemployment (2.00, 95% CI 1.42–2.82), low social activity participation (1.49, 95% CI 1.12–1.99), and discrimination (1.47, 95% CI 1.08–1.99) still had longitudinal associations with depressive symptoms in the adjusted model. For sensory/speech disability, employment status, occupational ability, social activity participation, discrimination, and leisure satisfaction had longitudinal associations with depressive symptoms in the crude model. After adjustment, unemployment (2.22, 95% CI 1.29–3.83), discrimination (2.66, 95% CI 1.70–4.17), and those not satisfied with leisure (1.90, 95% CI 1.19–3.04) had longitudinal associations with depressive symptoms. For physical-internal disability, employment status, occupational ability, assistance in daily living, and friendship satisfaction had a longitudinal association with depressive symptoms in the crude model. In the adjusted model, disability by disease (0.28, 95% CI 0.11–0.76), disability by unknown cause (0.19, 95% CI 0.05–0.67), loss of occupational ability (5.96, 95% CI 1.85–19.25) and dissatisfaction with friendship (2.45, 95% CI 1.29–4.67) showed longitudinal associations with depressive symptoms. For mental disability, cause of disability and employment status were the only two significant variables in both the crude and adjusted models. The crude HRs were 8.47 (95% CI 3.48–20.62) for disability by accident/industrial disaster, 3.61 (95% CI 1.52–8.59) for disability by disease, and 2.62 (95% CI 1.10–6.22) for disability by unknown cause. After adjustments, the HRs were 6.27 (95% CI 2.41–16.34) for accident/industrial disaster, 3.34 (95% CI 1.34–8.33) for disease, and 2.58 (1.04–6.44) for unknown cause. The crude HR for unemployment was 2.76 (95% CI 1.26–6.08), which increased to 2.81 (95% CI 1.14–6.94) after adjustment.

**Table 3:** Stratification analysis of two-year follow up of crude and adjusted hazard ratios (95% CI) for depressive symptoms by four types of disability.

	Physical-external		Sensory/speech		Physical-internal		Mental	
	Crude	Adjusted^†^	Crude	Adjusted^†^	Crude	Adjusted^†^	Crude	Adjusted^†^
**Cause of disability**								
Congenital/intranatal cause	1.00	1.00	1.00	1.00	1.00	1.00	1.00	1.00
Accident/ industrial disaster	0.47 (0.32–0.67)*	0.70 (0.47–1.07)	0.95 (0.50–1.81)	1.08 (0.55–2.13)	0.67 (0.17–2.59)	0.21 (0.04–1.04)	8.47 (3.48–20.62)*	6.27 (2.41–16.34)*
Disease	0.68 (0.46–0.99)*	0.82 (0.54–1.24)	1.29 (0.74–2.26)	1.48 (0.82–2.66)	0.60 (0.27–1.35)	0.28 (0.11–0.76)*	3.61 (1.52–8.59)*	3.34 (1.34–8.33)*
Unknown cause	0.89 (0.54–1.48)	1.05 (0.62–1.79)	1.55 (0.84–2.87)	1.73 (0.92–3.28)	0.54 (0.19–1.48)	0.19 (0.05–0.67)*	2.62 (1.10–6.22)*	2.58 (1.04–6.44)*
**Employment status**								
Employed	1.00	1.00	1.00	1.00	1.00	1.00	1.00	1.00
Unemployed	3.11 (2.38–4.05)*	2.00 (1.42–2.82)*	2.48 (1.64–3.73)*	2.22 (1.29–3.83)*	2.79 (1.46–5.32)*	1.07 (0.43–2.66)	2.76 (1.26–6.08)*	2.81 (1.14–6.94)*
**Occupational ability**								
Normal	1.00	1.00	1.00	1.00	1.00	1.00	1.00	1.00
Lower but able to work	1.81 (1.32–2.48)*	0.96 (0.67–1.38)	1.74 (1.08–2.82)*	1.19 (0.69–2.05)	1.93 (0.90–4.12)	2.18 (0.84–5.63)	1.47 (0.70–3.09)	1.09 (0.46–2.55)
Unable to work	2.88 (2.14–3.89)*	0.92 (0.61–1.37)	2.76 (1.71–4.46)*	1.49 (0.79–2.85)	4.28 (2.09–8.74)*	5.96 (1.85–19.25)*	1.22 (0.59–2.51)	0.64 (0.25–1.60)
**Social activity participation**								
High	1.00	1.00	1.00	1.00	1.00	1.00	1.00	1.00
Low	2.34 (1.80–3.03)*	1.49 (1.12–1.99)*	1.88 (1.25–2.81)*	1.51 (0.96–2.37)	1.67 (0.96–2.92)	0.69 (0.36–1.34)	1.33 (0.72–2.45)	1.03 (0.52–2.05)
**Assistance in daily living**								
Not/rarely needed	1.00	1.00	1.00	1.00	1.00	1.00	1.00	1.00
Needed	1.89 (1.46–2.46)*	1.09 (0.80–1.49)	1.32 (0.87–2.00)	0.76 (0.47–1.24)	1.86 (1.07–3.24)*	1.01 (0.50–2.06)	1.18 (0.71–1.97)	1.49 (0.80–2.78)
**Discrimination due to disability**								
Rare	1.00	1.00	1.00	1.00	1.00	1.00	1.00	1.00
Common	2.15 (1.63–2.84)*	1.47 (1.08–1.99)*	2.88 (1.92–4.30)*	2.66 (1.70–4.17)*	1.86 (0.93–3.71)	1.60 (0.71–3.64)	1.45 (0.87–2.41)	1.36 (0.78–2.35)
**Friendship satisfaction**								
Satisfied	1.00	1.00	1.00	1.00	1.00	1.00	1.00	1.00
Not satisfied	2.13 (1.64–2.75)*	1.19 (0.89–1.59)	1.39 (0.92- 2.11)	0.74 (0.46–1.17)	2.84 (1.64–4.90)*	2.45 (1.29–4.67)*	1.84 (1.04–3.25)	1.62 (0.81–3.24)
**Leisure satisfaction**								
Satisfied	1.00	1.00	1.00	1.00	1.00	1.00	1.00	1.00
Not satisfied	1.80 (1.35–2.40)*	1.32 (0.97–1.81)	1.76 (1.14–2.72)*	1.90 (1.19–3.04)*	2.03 (1.06–3.87)	2.15 (0.91–5.09)	1.29 (0.74–2.24)	0.80 (0.42–1.54)

^†^Adjusted for type of disability, cause of disability, gender, age, marital status, household income, household size, area of residence, employment status, occupational ability, social activity participation, assistance in daily living, discrimination due to disability, friendship satisfaction, leisure satisfaction, chronic conditions, and smoking. During each stratification analysis, factors that were stratified for were not included in the adjustment.

## Discussion

The current study suggests that significant causal relationship between disability and depressive symptoms and demonstrates different effect on depressive symptoms according to type of disability.

Looking at disability as a whole, factors associated with the risk of depressive symptoms after adjustment were the cause of disability, employment status, social activity participation, assistance in daily living, discrimination, friendship satisfaction, and leisure satisfaction. In the two-year follow-up, however, only the type of disability (physical-internal disability and mental disability), employment status, social activity participation, and discrimination were found to have an association with depressive symptoms. In short, being employed, participating in social activities, and not experiencing discrimination may contribute to reducing the risk of depressive symptoms in persons with disabilities in general.

Current analysis could demonstrate that associated factors with depressive symptoms significantly differ depending on disability types. The only common feature shared in all four disability types was that maintaining employment consistently decreased the risk of depressive symptoms.

In relation to occupational factors, employment was the key influential factor for depressive symptoms in physical-external disability, sensory/speech disability, and mental disability, while occupational ability was the key factor in physical-internal disability. Those who were unemployed showed more than twice the prevalence of depressive symptoms than those who were employed with a physical-external disability (2.00, 95% CI 1.42–2.82), sensory/speech disability (2.22, 95% CI 1.29–3.83), and mental disability (2.81, 95% CI 1.14–6.94), but not a physical-internal disability. In physical-internal disability, those unable to work showed remarkably higher odds (5.96, 95% CI 1.85–19.25) for depressive symptoms than those with normal ability to work. Conversely, in physical-external disability and sensory/speech disability, the relationships between inability to work and depressive symptoms disappeared after adjustment. In mental disability, occupational ability was not related to the risk of depressive symptoms both before and after adjustment. To synthesize, employment status could play a confounding or mediating role in relationships between other factors and depressive symptoms in physical-external, sensory/speech, and mental disabilities. In contrast, in physical-internal disability, occupational ability seems to function as a confounder or mediator between other factors and depressive symptoms.

Although this study is not informative regarding the mechanism, previous research can provide insights on such differences with regard to the impact of occupational factors by the types of disability. Zissi et al. demonstrated that employers’ attitudes and perceptions differed depending on the types of disability, with comparatively favorable attitudes toward physical-internal disability. There is also ample evidence that persons with physical disability are less likely to experience attitudinal barriers or stigma than persons with mental disability^13–17^. Moreover, visibility of disability affects the outside observers’ attitudes toward the persons with disability^13^. Although persons with visible disability were more likely to receive assistance from others^13,18^, this noticeability might also adversely affect employment. With this, persons with physical-internal disability—a physical and invisible form of disability—are the least likely to be deprived of employment opportunities due to negative prejudice or stigma despite normal ability to work. On the other hand, persons with physical-internal disability are more likely to leave the workforce due to the time and physical burden imposed by their disability. For instance, hemodialysis patients with renal failure who were employed faced difficulties in scheduling dialysis around work and in performing certain tasks due to general weakness or fatigue^19^. Likewise, although persons with physical-internal disability are relatively free from employers’ attitude-based challenges, their reduced ability to meet job responsibilities may change their occupational status, thereby affecting many other factors as well as psychosocial wellbeing. Meanwhile, for persons with other types of disability, especially mental disability, obtaining opportunities for employment per se may matter more than the ability to work.

With regard to independence, the relationship between the need of assistance in daily living and depressive symptoms seemed to be affected by other factors. With regard to physical-external disability and physical-internal disability, the relationship between the need of assistance in daily living and depressive symptoms disappeared after adjustment. Interestingly, for sensory/speech disability, those in need of assistance were found to have lower odds for depressive symptoms after adjustment, although this was not statistically significant (0.76, 95% CI 0.47–1.24). These results contrast with prior studies that suggested loss of independence in daily living as the key determinant of depression in physical disability^6,20,21^. In mental disability, there was no association between the need of assistance and depressive symptoms both before and after adjustment.

In relation to the factors associated with social inclusion and pursuit, the predictors of depressive symptoms were found to differ depending on the types of disability. In physical-external disability, low social activity participation and discrimination predicted the risk of depressive symptoms. In sensory/speech disability, low leisure satisfaction and discrimination predicted the risk. Considering that the HR of low leisure satisfaction elevated after adjustment, enjoyment of leisure seemed to effect on the relationships between other factors and depressive symptoms in sensory/speech disability. In physical-internal disability, only friendship satisfaction predicted depressive symptoms; and in mental disability, none of the following predicted depressive symptoms: social activity participation, discrimination friendship satisfaction and leisure satisfaction.

This may imply that persons with physical-external disability or sensory/speech disability may be either more exposed or more vulnerable to discrimination compared to physical-internal disability or mental disability. Tam et al. suggested that persons with visible disability are likely to develop less positive self-concept on account of social discrimination^22^. In light of this, it can be inferred that the noticeability of disability in combination with unsupportive views, prejudice, and stigma may lead to discrimination^23^. However, considering that an experience of disability discrimination may increase the odds for psychological distress^24^, it is still unclear why discrimination was not related to depressive symptoms in mental disability.

In relation to the cause of disability, it was widely believed that persons with congenital disability would adapt better than persons with acquired disability, although there have been few studies that verified this assumption^18^. The results of this study revealed that it may differ depending on the type of disability. The cause of disability was found to predict depressive symptoms, but the effects were the opposite in physical-internal disability and mental disability. After adjustment, in physical-internal disability, acquired causes such as disease (0.28, 95% CI 0.11–0.76) or accident or industrial disaster (0.21, 95% CI 0.04–1.04) showed significantly reduced HR for depressive symptoms compared to the congenital/intranatal cause. On the contrary, in mental disability, acquired causes of disability showed significantly higher HR for depressive symptoms compared to congenital/intranatal causes. Persons with mental disability by accident/industrial disaster, in particular, showed 6.27 times higher HR (95% CI 2.41–16.34) for depressive symptoms than those with congenital/intranatal causes. In physical-external disability, acquired causes seemed to predict reduced depressive symptoms before adjustment; however, this result was not statistically valid after adjustment. In sensory/speech disability, causes of disability were found to have no association with depressive symptoms.

In summary, the strongest predictor for depressive symptoms in mental disability was found to be the cause of disability—especially if it was an accident or industrial disaster. In contrast, the congenital/intranatal cause was found to be a strong predictor for depressive symptoms in physical-internal disability. Therefore, it seems that special monitoring and interventions are required to address the elevated risk of depression in the treatment of persons who acquired mental disability from accident or industrial disaster, and persons with congenital physical-internal disability. Among occupational or environmental factors, maintaining employment may be an effective way to reduce the risk of depressive symptoms in persons with mental disability. In contrast, retaining normal ability to work or professional function may be more important than employment status to reduce the risk of depressive symptoms in physical-internal disability.

## Limitations

Some limitations need to be considered when interpreting the results. Although the question used to assess depressive symptoms partly reflects the definition of depressive disorder, the clinical correlation between depressive symptoms and depression in this study is not ensured. In addition, participants who reported depressive symptoms at baseline were excluded from the survival analysis. Thus, there is a possibility that participants with long severe form of chronic depression were excluded from the survival analysis. We also do not know the reason for the follow-up loss of almost 19% of the initial participants, who were excluded from the baseline due to the missing data. However, severe depression may be one of the possible reasons of follow-up loss in such studies. Thus, considering the possibility that participants with severe depression might have been excluded from the analyses, the results of this study are not completely free from selection bias. Although we calculate length of time to event during followed up period, the event time did not correctly represent incident or event time because we used panel study design. For example, even though participant fall into depressive symptoms in different day to panel survey date, the event date is defined as same day to panel survey date. Hence, more detailed study design was needed to elucidate the time to event causality analysis. Our current study gathered participant using a two-stage cluster sampling survey method. Therefore, the prevalence and incidence should be calculated according to survey method using weighting values or consider variance. Hence, the amount of prevalence and incidence of depressive symptoms in the current study are not correct. Furthermore, individual participant is not completely independent to each other, due to the nature of two-stage cluster sampling survey method. Although, we did stratification analysis about sampling stratum, the OR and HR across disability types and economic activity status might not be generalized to total population.

## Conclusion

Through this two-year follow-up study with stratification by four types of disability, we demonstrated that the determinants of depressive symptoms differ, and the way those determinants impact may be varied—sometimes even the opposite—depending on the types of disability. Such diversity and heterogeneity of disability needs to be further evaluated and reflected into clinical and policy-level interventions to address the psychiatric needs of persons with different types of disability.

## Methods

### Study population and data collection

This study used data from the second wave of the Panel Survey of Employment for the Disabled (PSED). The PSED is a national open database which provides approved statistics (No. 383003) on South Korea and is administered by the Employment Development Institute, South Korea. The survey recruited registered disabled persons through the Welfare of Disabled Persons Act of South Korea residing in South Korea as of 2016. This cohort initially enrolled 4577 participants aged 15–64 years, which represent the working age population. The sample distribution and extraction were conducted using a two-phase sampling method which considered area of residence in the first phase and type and grade of disability, gender, age, and economic activity status in the second phase.

Participants selected in the cohort were interviewed every year from 2016 to 2018 with the same questionnaires based on the Tablet PC-assisted personal interviewing method. Accordingly, three cross-sectional datasets of the same cohort were collected to compose the three-year panel data. The participants who were lost to follow-up in 2017 and 2018 were excluded. However, it is assumed that the difference between the characteristics of the participants excluded and the participants included in the analysis is uninfluential, as the distribution of drop-out participants was relatively balanced among sub-groups in key variables such as gender, age, employment status, and disability type.

**Figure 1:**
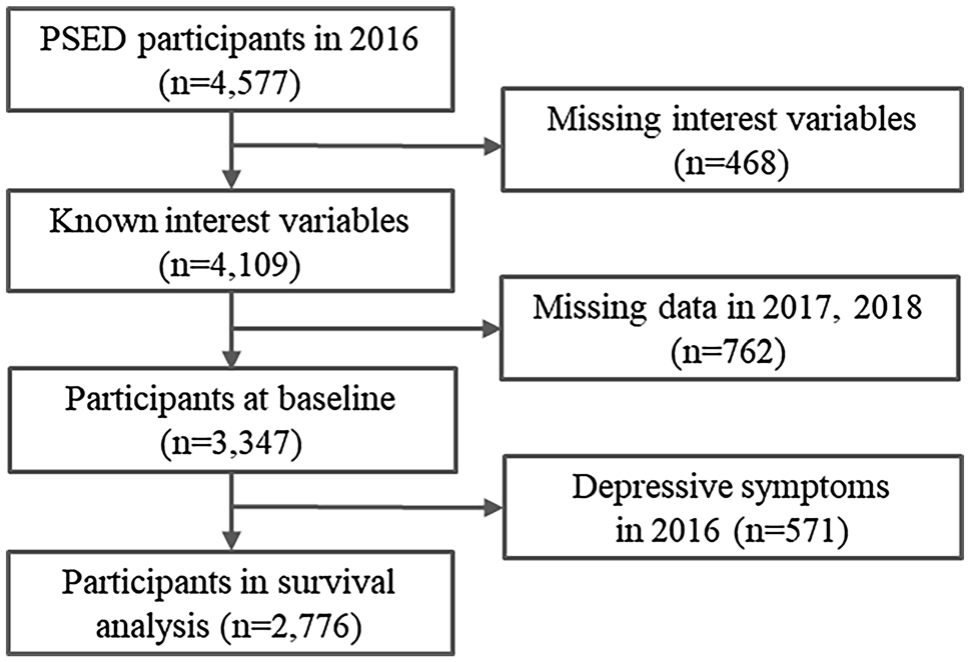
A schematic diagram of the study population.

For our study, among the initial 4577 participants of the 2016 PSED, 468 were excluded owing to missing data of interest variables (i.e., cause of disability, occupational ability, social activity participation, assistance in daily life, discrimination due to disability, friendship satisfaction, leisure satisfaction, chronic conditions, and smoking status). After the cross-sectional analysis of the 3347 participants at baseline in 2016, 571 participants were excluded for having depressive symptoms in 2016. Finally, a two-year prospective analysis was conducted on the 2776 participants without depressive symptoms at baseline. A schematic diagram of the study population is shown in Fig. 1.

### Variables

Type of disability was stratified by four categories: (1) physical-external, (2) sensory/speech, (3) physical-internal, and (4) mental. These reflect the official classification of disability types by the Welfare of Disabled Persons Act of South Korea^25^. According to this act, physical disability includes impairment of functions of major external body parts and disability of internal organs, while mental disability includes developmental disability and disability caused by mental disease. Under these four types of disability, a total of 15 sub-types were classified according to the aforementioned act: physical-external disability includes any physical disability, brain lesion, and facial disability; sensory/speech disability includes visual, auditory, and speech disability; physical-internal disability includes disorders in the kidney, heart, respiratory organs, and liver, intestinal/urinary fistula, and epilepsy; and mental disability includes intellectual, developmental, and psychiatric, as specified in the appendix of this article^25^. Psychiatric disability includes continuous bipolar affective disorder, schizophrenia, schizoid personality disorder, and recurrent depressive disorder that cause significant restrictions in daily or social lives.

Cause of disability was also categorized into four: (1) congenital/intranatal, (2) accident/industrial disaster, (3) disease, and (4) unknown cause. Disability by congenital or intranatal causes is characterized as a disability from the neonatal period or the very beginning of an individual’s life. Disability by accident or industrial disaster indicates that there was a specific event or an external factor which caused the disability. In contrast, disability by disease is caused by internal factors such as the person’s genetic factors or natural pathogenesis of an illness. Lastly, unknown cause is when the participant answered that he or she was not sure of the cause of their disability.

Socio-demographic variables included gender, age, marital status, household income, household size, and area of residence. Age was classified into 10-year intervals: < 35, 35–44, 45–54, > 55, resulting in four groups. Marital status was divided into two groups: living with partner (married/cohabiting) and without partner (single/divorced/widowed). Household income was grouped into four levels: < 10 million, 10–25 million, 25–40 million, and > 40 million Korean Won per annum. Household size (i.e., the number of family members who live together) was categorized into four levels: 1, 2, 3, 4, or more. Area of residence was divided into non-metropolitan (middle or small-sized town, which includes rural areas) and metropolitan according to the national classification of administrative areas.

Other variables represent contextual factors including occupational factors (employment status, occupational ability), independence (assistance in daily living), and social inclusion and pursuit (social activity participation, discrimination due to disability, friendship satisfaction, and leisure satisfaction). Employment status was categorized as employed or unemployed. Unemployed was defined as either losing a job or being economically inactive. Occupational ability (the ability to earn money by means of labor without considering any situational factors), was categorized into three levels: normal, lower but able to work, and unable to work. Social activity participation (a degree of participation in official and unofficial social meetings or gatherings and ceremonies such as weddings, funerals, graduations, and religious events) was grouped into high and low. Assistance in daily living (extent to which the respondent needs help from others in daily life) was grouped as not/rarely needed and needed. Discrimination due to disability (extent to which the respondent experiences disability discrimination in daily life) was grouped into rare and common. Friendship satisfaction was divided into two levels: satisfied and not satisfied, with the not satisfied response including both average and unsatisfied responses. Leisure satisfaction, including satisfaction with cultural and artistic pursuits, traveling, outdoor activities or religious activities, was grouped in the same way. Chronic conditions and current smoking status were classified into no/non-smoker or yes/current smoker.

Depressive symptomatology was assessed by a question regarding whether the participant had experienced feeling sad or hopeless severely enough to interfere with daily functioning for more than two weeks during the past year^26,27^. This single question may not be a diagnosed depression, used in screening depression in primary care and public health^28^.

### Statistical analysis

First, a cross-sectional analysis on the baseline participants was conducted to verify the association between variables and depressive symptoms. Afterwards, a two-year follow-up was conducted on participants without depressive symptoms at the baseline to investigate causality. In addition, both crude and adjusted models were presented to better understand the longitudinal relationships between variables and depressive symptoms. A multivariate logistic regression model was used to calculate odds ratio (OR) and 95% confidence interval (CI) for depressive symptoms in the baseline participants. Then a Cox proportional hazards model was used to calculate the hazard ratio (HR) and 95% CI for a two-year survival analysis on the participants without depressive symptoms at the baseline. In these analyses, crude (simple) and adjusted (disability type, cause of disability, gender, age, marital status, household income, household size, area of residence, employment status, occupational ability, social activity participation, assistance in daily living, discrimination due to disability, friendship satisfaction, leisure satisfaction, chronic conditions, and smoking) models were used. The length of time to event in Cox’s proportional hazard model were calculated between date of first survey event and date of observed day of depressive symptoms of “question regarding whether the participant had experienced feeling sad or hopeless severely enough to interfere with daily functioning for more than two weeks” For stratification analysis, participants without depressive symptoms at the baseline were divided into four categories for the disability type-specific survival analysis.

### Ethical statement

All PSED participants provided written informed consent for their voluntary participation. The current analysis was approved by the Institutional Review Board (IRB) of Yonsei University Health System (IRB No. Y2020-0169).

## Supplementary Information

Supplementary Information.
