# Supplementary material for: Predictors for depressive symptoms by four types of disability
Source: Sci Rep. 2021 Sep 29;11:19371. doi: 10.1038/s41598-021-98765-4 (PMC8481329; doi:10.1038/s41598-021-98765-4)
Supplement: Supplementary file 1 — Supplementary Information. [file 41598_2021_98765_MOESM1_ESM.docx]

**Title: Predictors for depressive symptoms by four types of disability**

**Appendix Table 1:** The prevalence of depressive symptoms of the participants at baseline by 15 sub-types of disability

| **Type of disability** | **Symptom (+)** | **No symptom** | **Total** |
| --- | --- | --- | --- |
| **Physical-External** | 385 (16.60%) | 1934 (83.40%) | 2319 (100.00%) |
| Physical | 320 (15.87%) | 1696 (84.13%) | 2016 (100.00%) |
| Brain lesion | 63 (21.72%) | 227 (78.28%) | 290 (100.00%) |
| Facial | 2 (15.38%) | 11 (84.62%) | 13 (100.00%) |
| **Sensory/ speech** | 136 (14.96%) | 773 (85.04%) | 909 (100.00%) |
| Visual | 91 (17.17%) | 439 (82.83%) | 530 (100.00%) |
| Auditory | 41 (12.77%) | 280 (87.23%) | 321 (100.00%) |
| Speech | 4 ( 6.90%) | 54 (93.10%) | 58 (100.00%) |
| **Physical-Internal** | 78 (23.78%) | 250 (76.22%) | 328 (100.00%) |
| Kidney | 43 (21.29%) | 159 (78.71%) | 202 (100.00%) |
| Heart | 1 ( 5.26%) | 18 (94.74%) | 19 (100.00%) |
| Respiratory | 3 (20.00%) | 12 (80.00%) | 15 (100.00%) |
| Liver | 7 (24.14%) | 22 (75.86%) | 29 (100.00%) |
| Intestinal/ urinary fistula | 5 (29.41%) | 12 (70.59%) | 17 (100.00%) |
| Epilepsy | 19 (41.30%) | 27 (58.70%) | 46 (100.00%) |
| **Mental** | 128 (23.15%) | 425 (76.85%) | 553 (100.00%) |
| Intellectual | 54 (15.04%) | 305 (84.96%) | 359 (100.00%) |
| Developmental | 4 (11.76%) | 30 (88.24%) | 34 (100.00%) |
| Psychiatric | 70 (43.75%) | 90 (56.25%) | 160 (100.00%) |

**Appendix Table 2**: General characteristics and the depressive symptoms of the participants of two-year follow up of the participants without depressive symptom in baseline.

|  | **Two-year follow-up of the participants**  **without depressive symptom** | | |
| --- | --- | --- | --- |
| **Variable** | **Symptom (+)** | **No symptom** | **Total** |
| **Type of disability** | (*P*-value) <0.001* | | |
| Physical-External | 238 (14.7) | 1375 (85.2) | 1613 (100.0) |
| Sensory/ speech | 98 (15.2) | 547 (84.8) | 645 (100.0) |
| Physical-Internal | 52 (25.0) | 156 (75.0) | 208 (100.0) |
| Mental | 61 (19.7) | 249 (80.3) | 310 (100.0) |
| **Cause of disability** | 0.002* | | |
| Congenital/ intranatal cause | 71 (16.9) | 349 (83.1) | 420 (100.0) |
| Accident/ industrial disaster | 136 (12.9) | 917 (87.1) | 1053 (100.0) |
| Disease | 168 (18.1) | 759 (81.9) | 927 (100.0) |
| Unknown cause | 74 (19.7) | 302 (80.3) | 376 (100.0) |
| **Gender** | 0.004* | | |
| Male | 266 (14.7) | 1545 (85.3) | 1811 (100.0) |
| Female | 183 (19.0) | 782 (81.0) | 965 (100.0) |
| **Age** | 0.17 | | |
| <35 | 123 (15.6) | 664 (84.4) | 787 (100.0) |
| 35-44 | 115 (14.5) | 677 (85.5) | 792 (100.0) |
| 45-54 | 126 (18.8) | 546 (81.3) | 672 (100.0) |
| >55 | 85 (16.2) | 440 (83.8) | 525 (100.0) |
| **Marital status** | <0.001* | | |
| Married/ cohabiting | 161 (11.8) | 1205 (88.2) | 1366 (100.0) |
| Single/ divorced/ widowed | 288 (20.4) | 1122 (79.6) | 1410 (100.0) |
| **Household income** | <0.001* | | |
| <10 | 188 (24.2) | 590 (75.8) | 778 (100.0) |
| 10-25 | 126 (16.9) | 620 (83.1) | 746 (100.0) |
| 25-40 | 65 (11.6) | 497 (88.4) | 562 (100.0) |
| >40 | 70 (10.1) | 620 (89.9) | 690 (100.0) |
| **Household size** | 0.001* | | |
| 1 | 108 (21.8) | 388 (78.2) | 496 (100.0) |
| 2 | 104 (15.8) | 555 (84.2) | 659 (100.0) |
| 3 | 107 (15.9) | 565 (84.1) | 672 (100.0) |
| 4 or more | 130 (13.7) | 819 (86.3) | 949 (100.0) |
| **Area of residence** | 0.49 | | |
| Non-metropolitan | 149 (15.5) | 815 (84.5) | 964 (100.0) |
| Metropolitan | 300 (16.6) | 1512 (83.4) | 1812 (100.0) |
| **Employment status** | <0.001* | | |
| Employed | 138 ( 9.2) | 1369 (90.8) | 1507 (100.0) |
| Unemployed | 311 (24.5) | 958 (75.5) | 1269 (100.0) |
| **Occupational ability** | <0.001* | | |
| Normal | 172 (11.0) | 1398 (89.0) | 1570 (100.0) |
| Lower but able to work | 128 (19.5) | 528 (80.5) | 656 (100.0) |
| Unable to work | 149 (27.1) | 401 (72.9) | 550 (100.0) |
| **Social activity participation** | <0.001* | | |
| High | 166 (11.2) | 1317 (88.8) | 1483 (100.0) |
| Low | 283 (21.9) | 1010 (78.1) | 1293 (100.0) |
| **Assistance in daily living** | <0.001* | | |
| Not/ rarely needed | 267 (13.8) | 1674 (86.2) | 1941 (100.0) |
| Needed | 182 (21.8) | 653 (78.2) | 835 (100.0) |
| **Discrimination due to disability** | <0.001* | | |
| Rare | 292 (13.5) | 1878 (86.5) | 2170 (100.0) |
| Common | 157 (25.9) | 449 (74.1) | 606 (100.0) |
| **Friendship satisfaction** | <0.001* | | |
| Satisfied | 240 (12.7) | 1652 (87.3) | 1892 (100.0) |
| Not satisfied | 209 (23.6) | 675 (76.4) | 884 (100.0) |
| **Leisure satisfaction** | <0.001* | | |
| Satisfied | 122 (11.4) | 945 (88.6) | 1067 (100.0) |
| Not satisfied | 327 (19.1) | 1382 (80.9) | 1709 (100.0) |
| **Chronic conditions** | <0.001* | | |
| No | 306 (14.4) | 1819 (85.6) | 2125 (100.0) |
| Yes | 143 (22.0) | 508 (78.0) | 651 (100.0) |
| **Smoking** | 0.1 | | |
| Non-smoker | 358 (16.2) | 1852 (83.8) | 2210 (100.0) |
| Current smoker | 91 (16.1) | 475 (83.9) | 566 (100.0) |

**Appendix Table 3**: A cross-sectional (at baseline of total participants) and a longitudinal (two-year follow up of participants without depressive symptom) analysis on the crude and adjusted hazard ratios (95% CI) for depressive symptoms.

|  |  | |  | |
| --- | --- | --- | --- | --- |
|  | **Cross-sectional analysis of the total subjects (1st year)** | | **Two year follow-up of the subjects with no depressive symptom** | |
|  | **Crude** | **Adjusted†** | **Crude** | **Adjusted†** |
| **Type of disability** | | | | |
| Physical-External | 1.00 | 1.00 | 1.00 | 1.00 |
| Sensory/ speech | 0.88 (0.71-1.09) | 0.83 (0.67-1.08) | 1.04 (0.82-1.31) | 0.98 (0.77-1.25) |
| Physical-Internal | 1.57 (1.18-2.06)* | 1.24 (0.90-1.71) | 1.78 (1.32-2.41)* | 1.39 (1.01-1.92)* |
| Mental | 1.51 (1.20-1.89)* | 0.80 (0.60-1.06) | 1.37 (1.04-1.82)* | 0.69 (0.50-0.95)* |
| **Cause of disability** | | | | |
| Congenital/ intranatal cause | 1.00 | 1.00 | 1.00 | 1.00 |
| Accident/ industrial disaster | 1.31 (1.00-1.74) | 1.59 (1.16-2.20)* | 0.75 (0.56-1.00)* | 1.01 (0.74-1.39) |
| Disease | 1.75 (1.33-2.31)* | 1.68 (1.24-2.30)* | 1.09 (0.82-1.43) | 1.15 (0.85-1.54) |
| Unknown cause | 2.01 (1.48-2.74 )* | 1.80 (1.30-2.52)* | 1.19 (0.86-1.65) | 1.16 (0.83-1.62) |
| **Employment status** | | | | |
| Employed | 1.00 | 1.00 | 1.00 | 1.00 |
| Unemployed | 2.84 (2.39-3.38)* | 1.34 (1.05-1.71)* | 2.93 ( 2.39-3.58)* | 1.99 (1.54-2.57)* |
| **Occupational ability** | | | | |
| Normal | 1.00 | 1.00 | 1.00 | 1.00 |
| Lower but able to work | 2.06 (1.67-2.53)* | 1.03 (0.80-1.32) | 1.87 (1.49-2.35)* | 1.09 (0.84-1.41) |
| Unable to work | 3.84 (3.17-4.67)* | 1.19 (0.89-1.58) | 2.71 (2.17-3.37)* | 1.08 (0.80-1.45) |
| **Social activity participation** | | | | |
| High | 1.00 | 1.00 | 1.00 | 1.00 |
| Low | 3.14 (2.63-3.76)* | 1.65 (1.35-2.03)* | 2.07 (1.71-2.51)* | 1.38 (1.12-1.71)* |
| **Assistance in daily living** | | | | |
| Not/ rarely needed | 1.00 | 1.00 | 1.00 | 1.00 |
| Needed | 2.18 (1.86-2.57)* | 1.34 (1.10-1.64)* | 1.66 (1.38-2.01)* | 1.03 (0.83-1.28) |
| **Discrimination due to disability** | | | | |
| Rare | 1.00 | 1.00 | 1.00 | 1.00 |
| Common | 2.42 (2.04-2.86)* | 1.67 (1.37-2.03)* | 2.10 (1.73-2.55)* | 1.69 (1.36-2.09)* |
| **Friendship satisfaction** | | | | |
| Satisfied | 1.00 | 1.00 | 1.00 | 1.00 |
| Not satisfied | 3.75 (3.17-4.44)* | 1.89 (1.55-2.30)* | 2.00 (1.66-2.40)* | 1.20 (0.97-1.48) |
| **Leisure satisfaction** | | | | |
| Satisfied | 1.00 | 1.00 | 1.00 | 1.00 |
| Not satisfied | 3.01 (2.46-3.71)* | 1.72 (1.37-2.16)* | 1.75 (1.42-2.16)* | 1.35 (1.08-1.69)* |

†Adjusted for type of disability, cause of disability, gender, age, marital status, household income, household size, area of residence, employment status, occupational ability, social activity participation, assistance in daily living, discrimination due to disability, friendship satisfaction, leisure satisfaction, chronic conditions, and smoking. During each stratification analysis, factors that were stratified for were not included in the adjustment.
